# Correction to: Lenihan, et al., MDRD-Estimated GFR at One Year Post-Renal Transplant is a Predictor of Long-Term Graft Function

**DOI:** 10.1080/0886022X.2018.1429240

**Published:** 2018-01-24

Lenihan CR, O’Kelly P, Mohan P, et al. MDRD-Estimated GFR at One Year Post-Renal Transplant is a Predictor of Long-Term Graft Function. Renal Failure. 2009;30:345–352. https://doi.org/10.1080/08860220801947686.

When the above article was published online and in print, wrong information was used regarding the first renal transplant in Ireland. In recent times we have been made aware that in fact it took place in December 1963 at St. Vincent’s Hospital in Dublin.

The authors apologize for this error.

